# Potential through simplicity: thymidine kinase-1 as a biomarker for CDK4/6 inhibitors

**DOI:** 10.1038/s41416-020-0858-y

**Published:** 2020-05-08

**Authors:** Amelia McCartney, Luca Malorni

**Affiliations:** 1 “Sandro Pitigliani” Medical Oncology Department, Hospital of Prato, Prato, Italy; 2 “Sandro Pitigliani” Translational Research Unit, Hospital of Prato, Prato, Italy

**Keywords:** Prognostic markers, Breast cancer, Translational research

## Abstract

We describe a potential role for thymidine kinase-1, a general marker of cellular proliferation, to act as a prognostic biomarker in patients receiving CDK4/6 inhibitors for advanced hormone receptor-positive, HER2-negative breast cancer, with early data suggesting that it may also provide early indication of treatment response.

## Main

Cancer is classically associated with cell cycle dysregulation, which is characterised by an evasion of the effect of growth suppressors and unchecked cellular proliferation. Progression within the cell cycle from the primary growth phase G1 to S phase (DNA synthesis) embodies a check point that protects against abnormal cellular replication. Progression from G1 to S is dependent on the cyclin-dependent kinases 4 and 6 (CDK4/6), which, under normal conditions, hyperphosphorylate and inactivate the retinoblastoma gene (Rb) product, which in turn allows the release and activation of the E2F family of transcription factors, resulting in successful progression to S phase. The adoption of CDK4/6 inhibitors into the management of breast cancer was underpinned by an awareness that alterations in the cyclin D1–CDK–Rb axis are associated with the development of hormone receptor (HR)-positive breast cancer, with CDK4/6 playing a pivotal role in driving malignant cell cycle progression. Exploiting this knowledge, three selective inhibitors of CDK4/6 (palbociclib, ribociclib, and abemaciclib) have been developed and trialled, demonstrating a significant and meaningful advantage in terms of progression-free survival (PFS) in patients with HR-positive, HER2-negative metastatic breast cancer, both in the first line of palliative treatment as well as in the later line setting, following exposure to previous endocrine therapy. There is additional emerging data that also suggest a benefit in terms of overall survival.

While the resoundingly positive results of the Phase 3 CDK4/6 landmark trials have established CDK4/6 inhibitors as standard of care for HR-positive, HER2-negative advanced disease, the only biomarker of any utility proven thus far is HR status. Approximately 10% of patients who receive a CDK4/6 inhibitor plus endocrine therapy as first-line treatment for metastatic breast cancer will exhibit primary resistance to the agent. Furthermore, progression on these drugs is considered inevitable, yet to date, clinicians have no predictive or prognostic biomarker of response to CDK4/6 inhibitors. Much of the early attempts at biomarker discovery for these agents adopted a repeated bias towards the hypothesis of a single, convergent pathway that might reveal an efficacious marker. A common intuitive assumption was that a predictive marker might be found within the cyclin D1–CDK–Rb axis. This premise was tested in the Phase 2 PALOMA-1 trial, which failed to show any role for the amplification of cyclin D1 or loss of p16 (INK4A—a negative regulator of the pathway) as predictive markers.^1^ Despite these initial negative results, numerous subsequent biomarker subanalyses of the landmark trials continued to circle this ground, with limited success.^2,3^ More recently, it has been shown that dysregulation of other signalling pathways, with or without common convergence on the cyclin D1–CDK–Rb axis, may contribute to the development of resistance to CDK4/6 inhibitors, suggesting multiple mechanisms operating in different subsets of patients.^4^ Such heterogeneity adds complexity to the task of a true personalisation of treatment in this field, which remains an area of intense research. However, a reliable biomarker able to provide a quick and flexible readout of the activity of this axis, irrespective of any knowledge of the driving molecular aberration, may fill the gap in this setting.

Thymidine kinase-1 (TK1) is a well-described, cell cycle-dependent cytosolic enzyme that plays a pivotal role in DNA synthesis and cellular proliferation. In resting cells, observable TK1 activity is low to absent, increasing during G1/S transcription, and peaking at S phase.^5^ In healthy subjects, levels of TK1 are low to absent, with contrastingly elevated levels observed in patients with a range of malignancies, including breast cancer.^6^ Notably, the synthesis of TK1 is regulated by the E2F pathway, making it an appealing potential marker for CDK4/6 inhibitors. Preclinically, we have shown that, among E2F target genes, TK1 was one of the most differentially expressed genes between acquired resistant (PDR) and sensitive (PDS) palbociclib-treated, oestrogen receptor-positive breast cancer cell lines. TK1 mRNA levels modulated in response to palbociclib treatment in PDS cell lines, but not in PDR cells.^7^ Furthermore, TK1 enzymatic activity was shown to decrease significantly earlier in response to palbociclib treatment than a corresponding reduction in cellular proliferation rate, suggesting that alterations in TK1 activity may serve as an early indicator of response to palbociclib. This hypothesis was tested by retrospectively quantifying TK1 activity in plasma collected prospectively at baseline, after one cycle of treatment, and at disease progression from patients enrolled in TREnd (NCT02549430), a Phase 2 study that compared the efficacy and safety of single-agent palbociclib versus palbociclib plus the endocrine therapy previously received for advanced breast cancer.^8^ Patients with a rise in TK1 activity after one cycle of treatment (approximately 4 weeks from commencing palbociclib) had a significantly worse median PFS compared with  patients with stable or decreased levels of activity (3.0 versus 9.0 months, respectively, *P* = 0.002).^7^ In addition, patients with high levels of TK1 activity at the point of progression on trial fared worse than those with low levels, irrespective of the next line of treatment received after exiting the study (median time to treatment failure, 2.9 versus 8.7 months, *P* = 0.05).

Measurement of a general proliferative marker as a proxy for disease activity is comparatively far simpler than seeking a marker that is intrinsically exclusive and personalised to the therapy prescribed, the individual receiving the treatment, and/or the molecular and genomic idiosyncrasies of the disease itself. In that sense, TK1 may not seem initially appealing or particularly sophisticated, in that its simplicity and potential generalisability across different tumour types does not represent the hallmark of a truly “personalised” marker that is unique to the biology of breast cancer or to its clinical management. However, unlike other current methodological approaches used in precision medicine to tailor therapies, TK1 activity can be monitored through a simple, peripheral blood draw and measured via comparatively affordable enzyme-linked immunosorbent assay-based assays. There is no requirement of invasive biopsies, nor the need for highly specialised technical expertise in its analysis. A rise in TK1 activity observed within weeks of commencing a CDK4/6 inhibitor—signifying ongoing cellular proliferation, in spite of the presence of a selective inhibitor—may provide an early warning that the treatment will fail to provide benefit. Furthermore, this signal may potentially predate any radiological or clinical sign of progression. Efforts are ongoing to discover a specific predictive biomarker for CDK4/6 inhibitor therapy; however, in the meantime, utilisation of a proliferative biomarker that is both prognostic and indicative of early response may also augment clinical management.

Further validation of TK1 as a biomarker in the setting of CDK4/6 inhibition is required. Previously, TK1 activity has been shown to be both prognostic and a marker of resistance to treatment in patients receiving endocrine therapy for advanced HR-positive, HER2-negative breast cancer.^9^ These findings were later validated in a retrospective subanalysis of women enrolled in the Phase 3 EFECT (exemestane versus fulvestrant) study,^10^ with multivariate analyses showing TK1 activity to be an independent marker of prognosis after adjustment for major factors, including the presence of visceral disease, study treatment received, number of metastatic sites, and existing sensitivity to aromatase inhibitors. Prospective utilisation of TK1 as a prognostic marker in CDK4/6 inhibitor therapy is ongoing in larger studies and is anticipated to build on the initial findings within TREnd^7^ and will potentially facilitate multivariate analyses that were not feasible to conduct in the TREnd subanalysis due to low subgroup numbers. PYTHIA (NCT02536742) is a single-arm Phase 2 biomarker discovery study that has recently completed accrual in women with molecularly characterised HR-positive, HER2-negative advanced disease. TK1 activity will be assessed at pretreatment baseline and on palbociclib plus fulvestrant therapy and correlated to clinical outcome. Similar analyses at baseline and on treatment will be conducted within BioItaLEE, a Phase 3b study in postmenopausal women receiving letrozole plus ribociclib as first-line management of metastatic disease (NCT03439046).

## Data Availability

Not applicable.
